# Computational Study of Drop-on-Demand Coaxial Electrohydrodynamic Jet and Printing Microdroplets

**DOI:** 10.3390/mi14040812

**Published:** 2023-04-02

**Authors:** Zeshan Abbas, Dazhi Wang, Liangkun Lu, Yikang Li, Changchang Pu, Xiangji Chen, Pengfei Xu, Shiwen Liang, Lingjie Kong, Bin Tang

**Affiliations:** 1 Key Laboratory for Micro/Nano Technology and System of Liaoning Province, Dalian University of Technology, Dalian 116024, China; hopenotout1214@mail.dlut.edu.cn (Z.A.); llk@mail.dlut.edu.cn (L.L.); liyikang@mail.dlut.edu.cn (Y.L.); pcc@mail.dlut.edu.cn (C.P.); xjxj@mail.dlut.edu.cn (X.C.); xupfupc@163.com (P.X.); 2 Ningbo Institute of Dalian University of Technology, Ningbo 315000, China; liangsw_nbi@dlut.edu.cn (S.L.); klingjie2023@163.com (L.K.); 3 State Key Laboratory of High-Performance Precision Manufacturing, Dalian University of Technology, Dalian 116024, China; 4 Institute of Electronic Engineering, CAEP, Mianyang 621900, China; john46311@hotmail.com

**Keywords:** phase field model, electrohydrodynamic jet, DoD CE-Jet, computational study, microdroplets

## Abstract

Currently, coaxial electrohydrodynamic jet (CE-Jet) printing is used as a promising technique for the alternative fabrication of drop-on-demand micro- and nanoscale structures without using a template. Therefore, this paper presents numerical simulation of the DoD CE-Jet process based on a phase field model. Titanium lead zirconate (PZT) and silicone oil were used to verify the numerical simulation and the experiments. The optimized working parameters (i.e., inner liquid flow velocity 150 m/s, pulse voltage 8.0 kV, external fluid velocity 250 m/s, print height 16 cm) were used to control the stability of the CE-Jet, avoiding the bulging effect during experimental study. Consequently, different sized microdroplets with a minimum diameter of ~5.5 µm were directly printed after the removal of the outer solution. The model is considered the easiest to implement and is powerful for the application of flexible printed electronics in advanced manufacturing technology.

## 1. Introduction

Modern manufacturing technology integrates new production techniques and machinery with information technology, microelectronics, and innovative organizational practices in the manufacturing process. Therefore, the electrohydrodynamic jet (E-Jet) is one of the significant methods for fabricating micro/nano-structures executing electrostatic and hydrodynamic boundary conditions [1]. The E-Jet printing process is very popular in MEMS devices due to its advantages of high resolution and wide material adaptability during this current span [2]. It has been widely used for manufacturing micro/sub-micro structures of electronic, polymer, and ceramic materials. There are various E-Jet setup devices used by many groups and it consists of a syringe pump, a nozzle (metal), a high voltage system, and a substrate. Many researchers have used the combined needle system (metal and quartz) to print microstructures on insulating substrates for the application of electronic devices [3]. The simulation models determine the stable and control morphology of the cone jet when many basic forces, i.e., normal electric force, tangential force, surface tension, gravity force, dielectric force, and viscous force, take the state of equilibrium [4]. Contemporary development in the combined needle system demonstrates the advantages of E-Jet printing on electronic devices [5]. This trend has brought about a change in E-Jet technology and extends to a coaxial needle system that has the ability to print multi-layer composite structures on different substrates, i.e., conductive, non-conductive, and silicon substrates [6,7,8,9].

Coaxial electrohydrodynamic jet printing is a promising technology for preparing micro/nanoparticles on different substrates based on the drop-on-demand method [10]. Moreover, the working principle of DoD CE-Jet is the use of different needles, i.e., inner and outer capillaries by regulating functional fluids from the syringe pump. The inner and outer functional fluids have different physical properties and are smoothly delivered to the coaxial needle. The high voltage generator is connected with the coaxial needle system which produces the electric field around the needle tip. Similarly, Loscertales et al. produced monodisperse compound droplets using the coaxial electrohydrodynamic atomization process for the encapsulation of micro/nano materials [11]. Sun et al. generated special compositions known as core-shell polymer nanofibers by the co-electrospinning method using PEO polymer material as the internal fluid and PSU as the external fluid [12]. The co-electrospinning method can be used for the fabrication of versatile structures and microdroplets with the advantages of high precision packaging, strong controllability, and simple process. During theoretical analysis, the CEA process reports that the flow rate of internal and external fluids has a significant effect on the size of the composite microdroplets [11]. On the basis of the difference in electrical relaxation time between the inner liquid and the outer liquid, the study introduced the concept of conducting fluid including internal direction and external direction. Lee et al. described a multidrug encapsulation technique by coaxial tri-capillary electrospray (ES) system which can synthesize monodisperse PLGA-coated particles containing multiple drugs in one step [13]. Chen et al. studied spraying modes in coaxial jet electrospray with the outer driving liquid and showed that the modes of coaxial atomization depend on the physical properties of the outer liquid.

In addition, the stability of the coaxial jet can be heightened by changing the flow rate of the inner and outer liquids; furthermore, it was determined that the coaxial nozzle-assisted E-Jet printing for microscale 3D cell-laden constructs is essential in biomedical device applications [14]. The process parameters, i.e., applied voltage, alginate feeding rate, stage moving speed, and calcium chloride feed rate, were systematically studied to stably print microscale hydrogel filaments with 2D/3D organizations [15]. Moreover, the study introduced the fabrication of micro-nanocapsules by a new electrospraying technique using coaxial E-Jet printing. Furthermore, the study reported the effects of various operating parameters on the size of microdroplets and printed the direct writing stretchable and conductive cables for strain sensor by using a coaxial printing method [16]. The study used liquid metal in the inner capillary and high viscosity PDMS was used in the outer shell [17]. Ahmad et al. studied the generation of multilayer structures for biomedical engineering applications using a new device with three coaxial needles and E-Jet flow. The work concluded that E-Jet flow is an important phenomenon for two-layer bubbles, closed porous fibers, and nanocapsules containing three-layer structures [9]. Furthermore, Xu et al. performed a finite element simulation of CEA using the computational fluid dynamics (CFD) model in Fluent [18]. The ability of the CFD model to predict the output of composite structures was verified by the experimental study. However, the numerical simulation of the CEA process contained only the outer Taylor cone profile, which was not consistent with the experimental phenomena. Therefore, the study determined that both inner and outer Taylor cone profiles simultaneously existed [4]. In addition, throughout the simulation work, the volume charge density should be mainly concentrated on the two interfaces consisting of air-outer liquid and inner-outer liquid interfaces since it determines the magnitude and direction of the electric forces as the electric field is concentrated around the needle tip. According to the literature review on DoD CE-Jet printing, the effect of electric charge was also rarely discussed in previous works. Moreover, the current simulation works generally focus on the formation of the CEA process. Additionally, the influence of the key parameters on DoD CE-Jet printing including applied voltage and liquid flow rate were infrequently discussed and indicated that the DoD CE-Jet method still has challenges in printing stable microstructures [19].

In our research group, Xiaojun et al. described a coaxial focused electrohydrodynamic jet (CFEJ) printing technique and printed direct writing nanoscale structures [8]. Moreover, the different nano cantilever beam structures were introduced on a silicone substrate. The numerical simulation of CFEJ was developed based on a three-phase flow of liquid–liquid–air model. Throughout simulation and experimental study, the PZT solution was used in the inner needle and highly viscous silicon oil was used in the outer needle. Then, nanostructures of various patterns with a diameter of 40 nm were directly printed on silicon substrate. The results indicated that the three-phase field method is suitable for CE-Jet printing on various substrates for the application of micro-nano devices. It is well-known that E-Jet printing technology has a great importance for micro-nano devices for electronic applications [20]. Recently, the direct writing method has been used to fabricate functional nanostructures by reducing needle diameter. Park et al. fabricated various structures (diameter 240 nm) using a needle with a size of 300 nm [21]. However, the production of sub-microns is very difficult using the DoD CE-Jet printing method and only a special solution can be used for this complicated case. In the DoD CE-Jet process, the diameter of the inner jet can be reduced to sub-micron or even nanoscale with the assistance of strong electrical force and viscous shearing force originating from the outer solution. The DoD CE-Jet has the potential to produce nanoscale structures with a relatively large-sized coaxial needle system. However, the use of special ink and significant coaxial needles make DoD CE-Jet practice much more complicated. Therefore, the use of a numerical modeling technique can significantly help to determine optimized parameters and working conditions for precise microscale printing.

In favor of the latest developments from our group, the main objective of this DoD CE-Jet approach is to simulate compound droplets deformation under influence of a strong external electric field around the needle tip. We performed a DoD CE-Jet printing simulation and experiment in order to print stable compound microdroplets by generating a micro-dripping regime on silicon substrate. For a ternary phase field model, the Navier–Stokes equation including surface tension, viscous shear force, electrical force, internal pressure, and gravitational force were applied to describe a laminar flow of both functional fluids. Furthermore, the appropriate three-phase field technique was used to trace interfaces of inner-outer solution and air-outer solution. Based on the hypothetical examination, the formation of DoD CE-Jet was studied using a three-phase flow model in COMSOL Multiphysics software. By maintaining a constant outer viscous fluid flow, the influence of external pulse voltage and internal fluid flow velocity were investigated on CE-Jet morphology and microdroplet size. The DoD CE-Jet simulation results at different dc pulse voltages and flow velocities were verified by droplet generation during experiments. Therefore, in the experimental section, optimized parameters were considered as substantial stable parameters. The inner diameter of the designed inner needle was as large as 180 μm and the outer diameter was 420 μm in both the simulation and experiments.

## 2. Printing System and Printing Process

### 2.1. Materials

Particular materials were selected for numerical simulation and experimental studies throughout this work. Similarly, lead zirconate titanate (PZT) and photoresist (AZ703) were chosen as the inner liquid and high viscosity silicone oil was regulated as the outer liquid. The photoresist has a red color that can be easily detected during the microdroplet printing process. PZT is a sensitive piezoelectric material based on metal oxide which was developed by scientists at Tokyo Institute of Technology around 1952. The purpose of photoresist material is to be used in conjunction with the inner liquid, making it an ideal material and likewise improving the visibility of the PZT solution for the examination of printing droplets. The PZT solution is one of the most widely used in M/NEMS devices due to its excellent piezoelectric properties [22]. The properties of photoresist AZ703 (Anzhi Electronic Materials, China) and silicone oil (Dow Corning Corporation, USA) were obtained from the suppliers. Wang et al. used a special PZT solution that was cautiously prepared in laboratory [17]. The viscosity of the PZT solution was measured using a rotary viscometer (NDJ-79, Shanghai Pingxuan Scientific Instrument in China). The surface tension of the PZT solution was measured using a droplet shape analyzer (DSA100, Kruss GmbH in Germany). The relative permittivity of PZT solution was obtained using a precision resistance analyzer (4294A, Agilent Technologies in USA). Moreover, for the outer liquid, silicone oil is non-toxic, colorless, and highly viscous, which is very helpful in the formation of stable DoD CE-Jet for the generation of microdroplets. The silicon substrate is used for printing droplet strings with a thickness of 0.5 mm. Table 1 presents the physical properties of inner and outer liquids used in the research.

### 2.2. Drop-on-Demand CE-Jet Printing Setup

**Figure 1:**
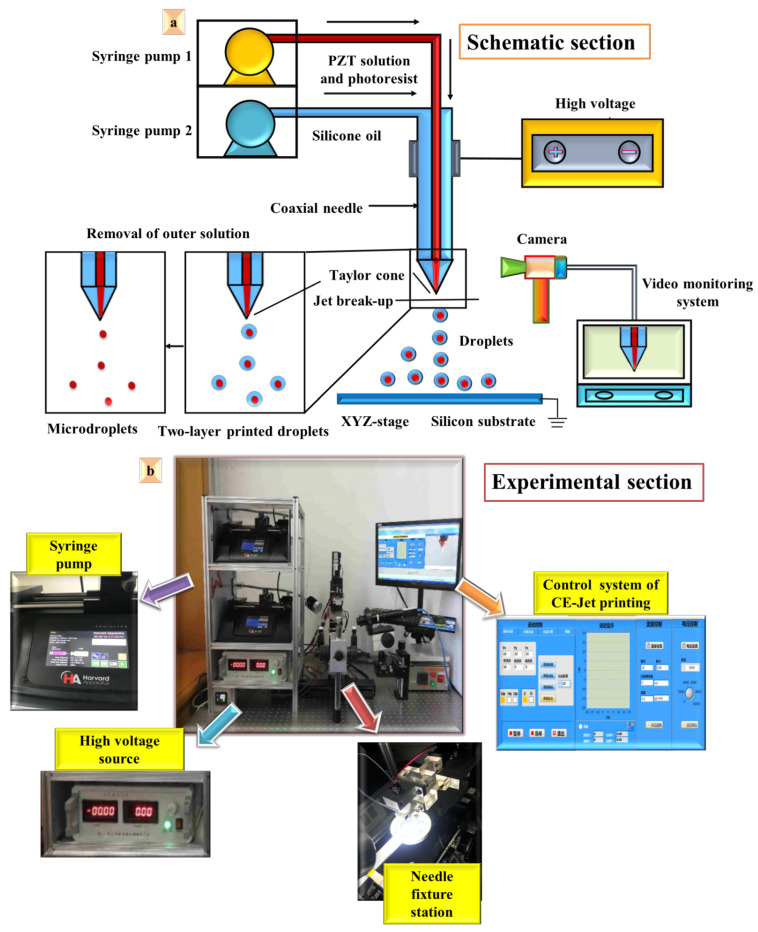
(**a**) Schematic diagram and (**b**) photograph of the DoD CE-Jet printing facilities.

In industrial coaxial E-Jet printing, there are two basic kinds of printheads (i.e., continuous E-Jet or direct writing heads and drop-on-demand (DoD) heads) used to print microdroplets for various electronic devices. Therefore, for engineered printing solutions, we need specialization in DoD CE-Jet printing machines. Similarly, Figure 1 illustrates the schematic and experimental setup of the DoD CE-Jet printing technique, which mainly consists of a coaxial needle, a high voltage dc pulse generator, two syringe pumps, a video monitoring system, and a computer-controlled XYZ motion stage. In the combined needle system, the inner and outer needles were respectively connected to the syringe pumps (PHD ULTRATM and Harvard Apparatus USA) by high-quality PTFE tubes. The inner needle was fixed inside the shell of the stainless-steel needle. In this work, the dimensions of the simulated geometric model are mainly based on commercial needles, in which the inner diameter of the inner needle is 180 µm and the outer diameter is 420 µm. Similarly, the outer needle has an inner diameter of 800 µm and the outer diameter is 1000 µm.

In addition, the coaxial needle system was connected to the high-voltage supply generator which is fundamentally a dc pulse source power supply. The electric field generated between the coaxial needle and the ground electrode produces electrical force which plays a key role in generating the Taylor cone profile at the needle interface. The silicon substrate, which was quite thick up to 0.5 mm, was placed at a distance of 16 cm from the needle tip. The silicon substrate was coated with Pt/Ti layers by using the sputtering technique in the lab. We measured the contact angle of the silicon substrate using an optical contact angle measuring instrument (KRUSS, Germany), which is exactly 65^o^. The angle is measured at less than 90^o^, which indicates that the silicon substrate has less polarizing effect of rising charges with high bonding force of the printed droplets on the substrate surface. It also has good wettability and durability in order to print high resolution drops. The flow rate values of the two functional solutions were adjusted and the unmixed liquids were injected at the appropriate flow rate using the two syringe pumps respectively. When a suitable voltage was applied, the surface tension force exceeded the gravity force of the liquid, and a stable DoD CE-Jet was formed at the needle tip. Subsequently, the micro-dripping mode appeared during the transition time of the cone profile and droplet disintegration, leading to the printing of droplet arrays on the substrate. After printing, a double-layered circle-encapsulated droplet with inner functional material and outer high viscous material was obtained. Then, by removing the external material, a microscale droplet of only internal functional materials can be obtained.

### 2.3. Numerical Simulation

#### 2.3.1. Governing Equations for Fluid Flow

**Figure 2:**
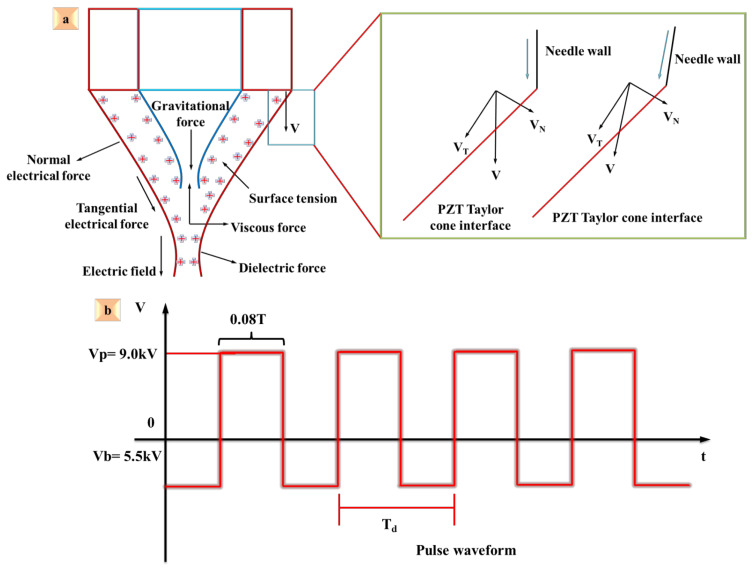
The effect of pulse waveform and analysis of different forces effect. (**a**) Forces acting around the needle tip in DoD coaxial E-Jet printing process. (**b**) The pulse waveform with different specific parameters such as base voltage, peak voltage, frequency, and duty cycle.

Three-phase flow in phase field method was based on the leaky dielectric model [12] and the Navier–Stokes–Cahn–Hilliard (NSCH) system [23]. In this work, three fluids (i.e., inner liquid, outer liquid, and air) are assumed to be non-miscible, incompressible, and Newtonian and laminar. The fluid flow can be defined in following continuity Equation (1):

(1) $$\nabla \vec{.u}=0$$

where $\vec{u}$ denotes liquid velocity vector of functional solutions. The high dc pulse voltage is applied to the coaxial needle system and free charges in both liquids produce electrical field polarization charges, which swiftly travel toward the three-phase interface. Consequently, the DoD CE-Jet, various fluid field interactions, and strong electric field are produced as a result of governing equations for fluid flow. In addition, as the electric field pushes charges around the coaxial needle interface, the inner and outer solutions are influenced due to electrical force meddling. Furthermore, the continuity and mass conservation of the Navier–Stokes equation for a three-phase system explain the forces acting around the needle tip during the DoD coaxial E-Jet printing process which is shown in Figure 2a. The momentum equation needs to include additional terms for surface tension on the needle interface, where F_st_ = [γ κ δ s n] and electric stress is F_es_; equations can be rewritten as in Equation (2). Moreover, Figure 2b illustrates the analysis of four parameters of pulse waveform in this work. The morphology of the cone jet is controlled in the experiments by adjusting four important parameters: peak voltage, base voltage, frequency, and duty ratio. The duty cycle is a single variable that ensures the process parameters are in constant position. The base voltage of 5.5 kV, peak voltage of 9.0 kV, printing speed of 100 mm/s, frequency of 100 Hz, and duty cycle of 80% were examined to develop a stable DoD CE-Jet printing process.

(2) $$\rho \frac{\partial \overset{}{u}}{\partial t}.(\overset{}{u}.\nabla ).\vec{u}=\nabla .\left[-\mathrm{pl}+\mu (\nabla \vec{u}+{\left(\nabla \vec{u}\right)}^{T}+F_{\mathrm{st}}+F_{\mathrm{es}}+\rho g\right]+F$$

where ρ is liquid density and p indicates internal pressure of the liquid. Similarly, F_st_ is surface tension force of liquid and F_es_ is electric force generated by electric field. The surface tension force is expressed by γ, κ and δ which are surface tension coefficient, curvature of interface, and Dirac-delta function, respectively. The viscous force tensor T is given in Equation (3), where μ is viscosity of liquids.

(3) $$T=\mu (\nabla \vec{u}+\nabla u^{T})$$

Evaluating variables and parameters in the phase field model is very exclusive, where a time-dependent factor plays a key role in tracing the ternary phase interfaces. Therefore, the three-phase field scheme was used to define the interface between the outer and inner phase in the simulation model. In order to account for the limitations of time, the Cahn Hilliard equations were formed in ternary phase field, which were identified in the software system [24,25] as given in Equation (4):

(4) $$\left\{ \begin{array}{l} \frac{\partial \phi_{i}}{\partial t}-\nabla .\vec{\mu }\phi_{i}=(\nabla .\frac{{Μ}_{o}}{\sum_{T}}).\Delta \eta_{i}\\ \eta_{i}=\frac{4\sum_{T}}{\theta }\sum_{i}\prod i\mp j-[\frac{1}{\sum_{j}}(\sigma_{i}F(\phi )-\sigma_{j}F(\phi ))](\frac{3}{4}-2\varepsilon c\Delta \theta \psi )\\ \eta_{A}=etaA,\eta_{B}=etaB \end{array}\right.$$

where σ expresses surface tension. Similarly, σ and ε are executed independently during Cahn Hilliard equations. Therefore, M_o_ is a driving force of liquids used as a variable and known as the diffusion coefficient. The concentration of two liquid phases during numerical simulation is very important to regulate fluid flow [26]. The Cahn Hilliard potential is an auxiliary variable which is used in variable contour to assist particle directions during the mass flow of liquids, as explained in Equation (5):

(5) $$\left\{ \begin{array}{l} F(\phi )=\sigma_{AB}\varphi_{A}{}^{2}\phi_{B}{}^{2}+\sigma_{AC}\phi_{A}{}^{2}\phi_{C}{}^{2}+\sigma_{BC}\phi_{B}{}^{2}\phi_{C}{}^{2}+\phi_{A}\phi_{B}\phi_{C}(\sum_{A}\phi_{A}+\sum_{B}\phi_{B}+\sum_{C}\phi_{C})\\ \sum_{i}=\sigma_{i,j}+\sigma_{i,k}-\sigma_{j,k} \end{array}\right.$$

where A, B, and C are phase variables used to identify location of each phase in three-phase flow model throughout simulation work. So, $\sum_{i}$ denotes total effect of surface tension [27]. The summation of entire three phases with high concentration values is assumed to be equal to unity in each numerical box, as given in the following Equation (6):

(6) $$\begin{array}{l} \varphi_{A}+\varphi_{B}+\varphi_{C}=1\\ \frac{3}{\sum_{i}}=\frac{1}{\sum_{A}}+\frac{1}{\sum_{B}}+\frac{1}{\sum_{C}} \end{array}$$

#### 2.3.2. Governing Equations for Interface Tracking

The Hamilton–Jacobi function which is given in Equation (7) is solved simultaneously with the Navier–Stokes equation during the three-phase field method, as given in the form of Equation (8) [28]:

(7) $$\begin{array}{l} \frac{\partial \alpha }{\partial t}+\nabla .(\cup .\alpha .\nabla )=0\\ Hence, \end{array}$$

(8) $$\alpha (x,t)=\left\{ \begin{array}{l} 0-----\mathrm{for}\to \Omega^{+}(Outerphase)\\ 1-----\mathrm{for}\to \Omega^{\_}(Innerphase)\\ 0\leq \alpha \leq 1-----\mathrm{for}\to T(Surface) \end{array}\right.$$

where α indicates phase fraction along with laminar flow of fluid and traces location of the interface around the coaxial needle tip. Similarly, Ω denotes the inner and outer phases of the PZT solution and silicon oil [29].

#### 2.3.3. Governing Equations for Electric Field

The Maxwell equations are approximated as electrostatic [30]. The magnetic effects are ignored, since the dynamic currents are in small quantities and in other words electric field is gyratory [31]. The electric field is expressed as in Equation (9):

(9) ∇×E=0

By applying Gauss’s law to a linear electric medium, it can be reduced to Equation (10):

(10) $$\nabla .(\varepsilon E)=\rho_{e}$$

where ε is permittivity and $\rho_{e}$ is a free charge density. Then, the free charge density is relative to current by charge conservation equation that dominates in system. Similarly, by using the electric charge approaches of Melcher and Saville for electric field, some of their work proposed density effect [32]. Thus, Issa presents Equation (11) which becomes the charge conservation equation in Ohmic regime for the immiscible liquids [33]:

(11) $$\frac{\delta \rho_{e}}{\delta_{t}}+\Delta .\rho_{e}u=-\nabla .(\varepsilon E)$$

To account for surface tension force F_st_, the continuum surface force was introduced as a volumetric force on the liquid-air interface, where it can be expressed as Equation (12) [34]:

(12) $${\vec{F}}_{st}=\sigma k(\nabla .\overset{\wedge }{n})$$

(13) $$F_{\varepsilon }=-\frac{1}{2}E^{2}\nabla \varepsilon$$

(14) $${\vec{F}}_{e}=q_{e}\vec{E}-\frac{1}{2}(\vec{E}.\vec{E}\nabla \varepsilon )$$

The relative permittivity of many materials can be considered constant, and the functional fluid is incompressible as given in Equation (13). Coulombic force is present in all the simplifications as given in Equation (14). This force always acts along the electric field that is perpendicular to the surface. The second term of the equation represents the permittivity gradient force, which is constant for each liquid and perpendicular to the surface.

### 2.4. Geometric Model Establishment and Physics Selection

The governing equations in the CE-Jet model describe viscous motion of a fluid, the expression of an electric field in a fluid, the tracking of an interface around a needle, and the coupling of flow field to the electric field. In this work, the CE-Jet is simulated to generate the micro-dripping mode for printing various micro and nano structures on a silicon substrate. An axisymmetric geometric model was established based on the actual experimental setup. In order to reduce the amount of calculation and improve simulation efficiency and accuracy of the simulation, the simulation model has been simplified in the following aspects:

(1) Converting a specific three-dimensional model into a two-dimensional axisymmetric model.

(2) Ignoring the outer diameter of the outer needle.

**Figure 3:**
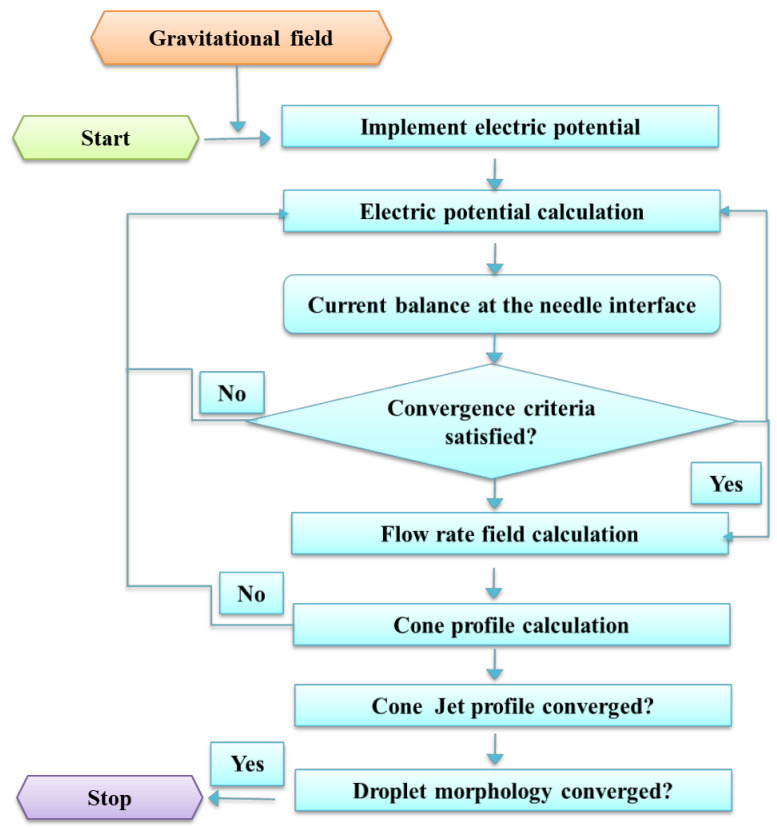
Flow diagram of the numerical simulation model.

**Figure 4:**
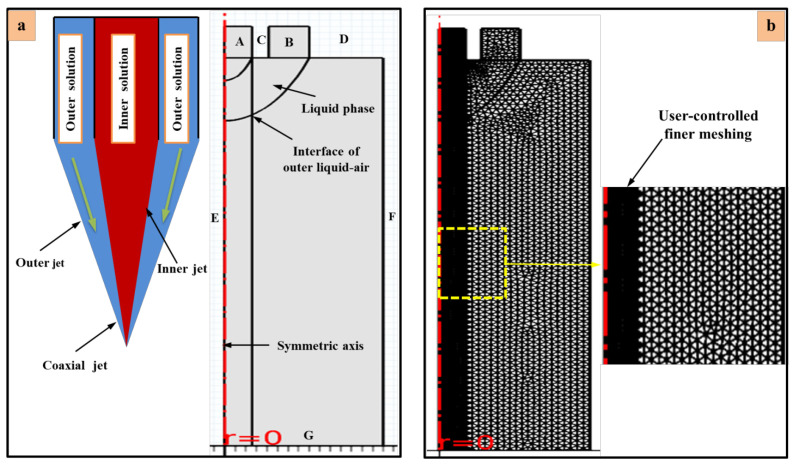
Numerical simulation of DoD CE-Jet printing. (**a**) The geometry of phase field model and particular boundary conditions. (**b**) The finer user-controlled meshing for model.

The reason for this is that needle shape is axisymmetric and two-dimensional axisymmetric model is sufficient to express the coaxial needle model. Figure 3 displays the flow diagram of the numerical simulation model. Furthermore, the simulation difficulty and calculation quantity can be greatly simplified, and the outer diameter of the needle has no influence on flow field. The geometric model consists of two different types of boundary conditions, one boundary line being constructed during the distribution of high electric field and another along the laminar flow of liquids at needle inlet. During the laminar flow in the physical model, the reference pressure level was chosen at 1 atm and reference temperature level was selected at 293.15 K, which determined multiple coupling options for smooth fluid flow. Similarly, Figure 4a shows the geometry of the phase field model and particular boundary conditions. The simulation model was constructed based on time-dependent unit, where the time range was (0, 5 × 10^−5^, 0.5). The results were resolved in 2D plot group using a relative tolerance value of 0.01. Similarly, the absolute tolerance value of 5 × 10^−4^ was used, which reinforces the consistent initialization of backward Euler value that was measured at 0.001. Figure 4b demonstrates finer user-controlled meshing for the inner needle and physics-controlled meshing for the remaining space.

Moreover, the combined effects of forces are attributed to three aspects of force, including gravity, electric field force, and fluid dynamics. Thus, it is necessary to introduce four physics fields, i.e., gravity fields, electrostatic fields, flow fields, and multiphysics that require coupling level. When the gravitational field is included in the flow field, the gravitational acceleration is set at 9.8 m s^−2^ and the direction is on the Y axis. Because the dielectric constant of the material jumps across the interface point, it must adjust the relative dielectric constant and space charge density in the electrostatic field. The boundary conditions of the geometric model are summarized in Table 2. The inner liquid generates inner CE-Jet under the high applied voltage and similarly the outer silicon oil produces outer CE-Jet at the same voltage. Here, φ is the initial value of pulse voltage, u is the fluid initial velocity, and V_0_ is the applied voltage to the coaxial needle. Furthermore, as the voltage varies from different locations and is determined in the form of V during the calculation, Q_inner_ is the flow rate of inner liquid and A_inner_ territory represents the cross-sectional area of the inner needle. Q_outer_ shows the flow rate of outer liquid and the territory of A_outer_ is the difference of cross-sectional area between the inner and outer needle.

The formation of DoD CE-Jet mainly depends on the flow of the outer liquid and the distribution of electric filed in the phase field method. Charge density of electric potential around the outer liquid affects the physical properties of silicon oil, which produces stable CE-Jet around the needle tip. Meanwhile, the CE-Jet is concentrated in the region close to the symmetric axis and converts to the micro-dripping mode to produce microdroplets. This is the change of electric field and the flow of fluid almost only occurred in the region around the axis of symmetry. In this situation, meshes located merely in the region approaching the axis of symmetry are required to be refined by applying the user-controlled sequence type, which can help to improve the efficiency and accuracy of the simulation.

## 3. Results and Discussion

### 3.1. Effect of Charge Density on Droplet Formation

**Figure 5:**
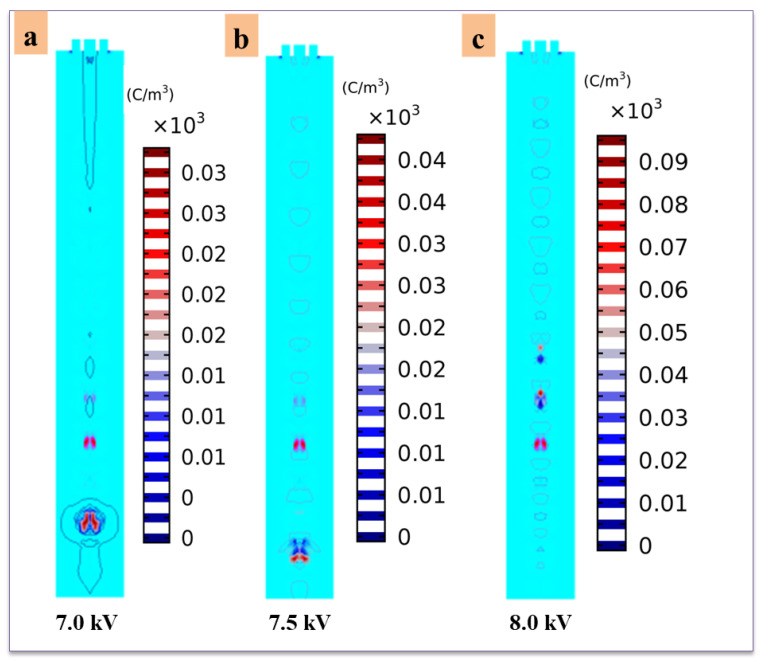
(**a**–**c**) are the space charge density profiles at the liquid and gas interface during microdroplets formation based on nozzle pulse voltages of 7.0, 7.5, and 8.0 kV, respectively.

Generating a stable cone jet profile for the micro-dripping mode in order to print microstructures on substrates using CE-Jet technique is necessary to evaluate and observe the concentration of space charge density around the needle interface. During the experiments performed in simulation, the charge distribution around the interface of the inner-outer solution and at equal times the distribution of charge around the interface of the outer-air solution was determined. The space charge density ranges from 0.02 to 1.02 C/m^3^ and most of the charges are accumulated at the cone apex. As mentioned in Figure 5a,b, light blue color with red spots indicates distribution of electrical charges around the coaxial needle interfaces over the duration of time (0.02 s). Similarly, as the time span moves forward (0.04–0.09 s), the space charge distribution becomes thick around the needle interface as shown in Figure 5c. The distribution of charges around the Y component produces a hyperbolic inverse effect, so that the distance between internal and external CE-Jets becomes larger, which causes formation of the micro-dripping mode. The territory around the tip of needle determines that abundant electrical charges are accumulated and break CE-Jet and turn it into a small droplet array leading to surface of substrate. However, the effect of charge density on droplet formation is uneven, which shows irregularity and non-uniformity in some drops generated in space.

The comparison at different applied voltages illustrates that maximum electrical charges are concentrated around the tip of the Taylor cone when the voltage gradually increases in electrostatic contour. Lastow et al. discussed the effect of volume charge distribution on the cone jet profile but the numerical simulation did not involve a droplet generation [35]. Alfonso and Gañán-Calvo introduced the phenomenon of steady tip streaming to explore high-quality sprays. The study discussed and investigated several jet regimes for the atomization process to extend and stabilize the cone jet [36]. Lyras et al. presented a conservative level set method for liquid-gas flows based on two-phase flow for the application of atomization process [37]. Zahoor et al. presented a numerical investigation to check the influence of gas type concentration on the characteristics of liquid micro-jets. The study was useful to investigate the shape and morphology of the nozzle cone jet evolution [38]. The numerical study discoursed that if the charge accumulates around the needle interface at a certain level, then electrical force begins to exceed the surface tension, which causes production of cone jet morphology. Furthermore, in the CE-Jet printing process some researchers introduced numerical simulation of droplet generation for pharmaceutical applications because studies on the stability of coaxial micro-jet in an electric field are still quite limited. Xu et al. executed numerical simulation of CEA process for production of polymeric composite microspheres under the effect of strong volume charge density [18]. Similarly, Yan et al. also introduced a numerical model which described core-shell droplet formation using CEA process by regulating high voltage [17]. However, the existing phase field model aimed to generate stable and controlled droplets by adjusting high dc positive pulse voltage that increases high concentration value of space charge density. Space charge density distribution photographs are taken at optimized dc positive pulse voltage, which is 8.0 kV in existing work.

### 3.2. Effect of Applied Voltage on Droplet Formation

**Figure 6:**
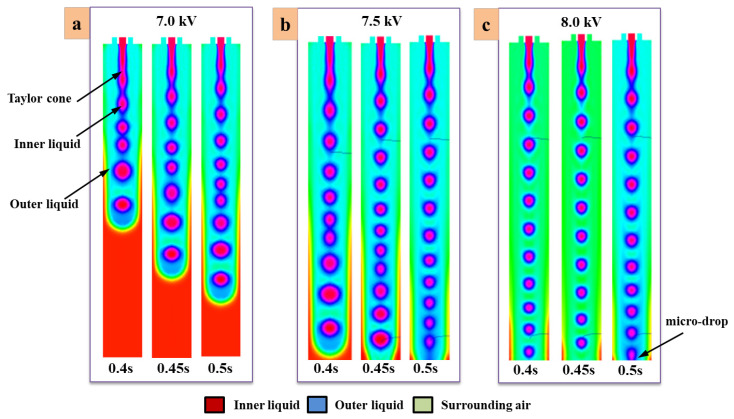
The formation of compound microdroplets during stable micro-dripping mode at different time intervals under various nozzle pulse voltages: (**a**) 7.0 kV, (**b**) 7.5 kV, and (**c**) 8.0 kV.

The high dc positive pulse voltage value is used to generate the electric field for DoD CE-Jet printing process and is considered a substantial and crucial parameter. The influence of electric field on the diameter of a droplet was examined by adjusting different values (5.5 to 9.0 kV) of dc pulse voltage. The print height and flow velocity of outer-inner liquid (internal fluid velocity 150 m/s, external fluid velocity 250 m/s, print height 16 cm) were kept constant during the simulation to control effect of voltage applied to droplet size. The simulation experiments results are presented at different voltages applied, respectively, 7.0 kV, 7.5 kV, and 8.0 kV. The red region is an inner solution defined as phase A, the light blue region is the outer liquid that is defined as phase B, and the green region is surrounding air defined as phase C. It can be seen from Figure 6 that as pulse voltage increases, the Taylor cone develops smaller and transforms to micro-dripping mode. Then, the droplet diameter turns out to be smaller in size. The CE-Jet equipotential lines are inversely proportional to the applied dc pulse voltage. This is mainly due to increase in voltage caused by tangential electric field acting on the cone jet profile.

In addition, Figure 6(a,b,c) illustrates different morphologies of the microdrop array at different values of pulse voltage. The electric field greatly influences micro-dripping mode by increasing applied voltage from 7.0 kV to 8.0 kV and the diameter of the droplet gradually decreases. Furthermore, the angle of cone profile between inner and outer needle increased over time, keeping other parameters constant. The rationale behind this phenomenon is that electric field strength increases with rising applied voltage around the interface, which can improve DoD CE-Jet actuation to generate droplet array. During the simulation, when the pulse voltage is too low at a value of 6.5 kV, then electric field strength is insufficient to overcome surface tension and viscous force. However, accumulation of liquid at the needle exit is greater than liquid sprayed on the substrate and CE-Jet formed at this time is coarser. Theoretically, when regulated voltage is high, this increases tangential electric field force at certain time intervals. Furthermore, small DoD CE-Jet diameter is obtained to print small-size microdroplets. Similarly, when the voltage is too high, then liquid spray on the substrate is larger in proportion to the actual amount than discharged liquid that can be supplied by the syringe pump. The CE-Jet breaks down at an applied pulse voltage of 8.0 kV near the interface of the needle and forms a hole of empty space with the micro-dripping mode. The simulation results showed that if voltage is further increased (more than value 8.0 kV), instability of CE-Jets may occur and even the breakdown of electric charges, causing instability in formation of the DoD CE-Jet. Similarly, for a needle voltage of 7.0 kV, a large amount of photoresist PZT solution is found circulating within the vortex (Figure 6a); moreover, that PZT solution propagates longer within the vortex. In contrast, for needle voltages of 7.5 and 8.0 kV, PZT solution no longer propagates and stays small within the vortex (Figure 6b and c). For all the three cases, the PZT solution is drawn towards CE-Jet formation and there is consistent formation of compound droplets. In the silicone oil case, it propagates longer inside the vortex, protecting the inner fluid in all three cases. The interfacial surface tension was defined at 0.07 N/m between the inner liquid and outer liquid. This interfacial surface tension was defined since the inner liquid does not contact the air phase directly but the silicone oil in the outer nozzle.

### 3.3. Effect of Flow Velocity on Droplet Formation

**Figure 7:**
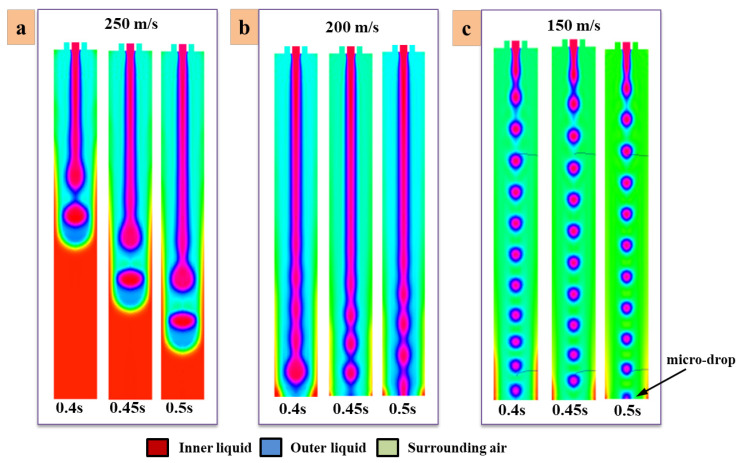
The formation of bilayer microdroplets during stable micro-dripping mode at different time intervals under different values of inner flow velocities (**a**) 250 m/s, (**b**) 200 m/s, and (**c**) 150 m/s.

Maintaining pulse voltage and print height constant (voltage 8.0 kV, external fluid velocity 250 m/s, print height 16 cm), several simulation experiments were performed in the range from 100 to 300 m/s. The simulation results are presented under different inner flow velocities (i.e., 250 m/s, 200 m/s, and 150 m/s) and at different time intervals for stable compound droplet formation as shown in Figure 7a–c. Similarly, the simulation results illustrate that the DoD CE-Jet is longer at higher flow velocity of the PZT solution. It converts to micro-dripping mode and forms large-size microdroplets, as can be seen from Figure 7a. As flow velocity of the inner PZT solution varies to 200 m/s, the size of the Taylor cone reduces and CE-Jet diameter changes significantly due to reduction of electric field outside the inner fluid vortex. The size of droplet and DoD CE-Jet is proportional to the flow velocity of the inner liquid. This situation is mainly due to increase in traffic flow of liquid particles, which is caused by unit time. The high flow velocity of PZT solution accumulates liquid molecules at the interface of the outer needle and produces an agglomeration effect, which makes the cone jet profile longer as shown in Figure 7b. Therefore, the droplet size increases, and the shape of the droplet becomes elliptical. Theoretically, the smaller the internal liquid flow velocity, the smaller the DoD CE-Jet diameter. In our case, the length of DoD CE-Jet is short, which transforms into the micro-dripping mode due to strong electric field effect to form stable compound microdroplets.

**Figure 8:**
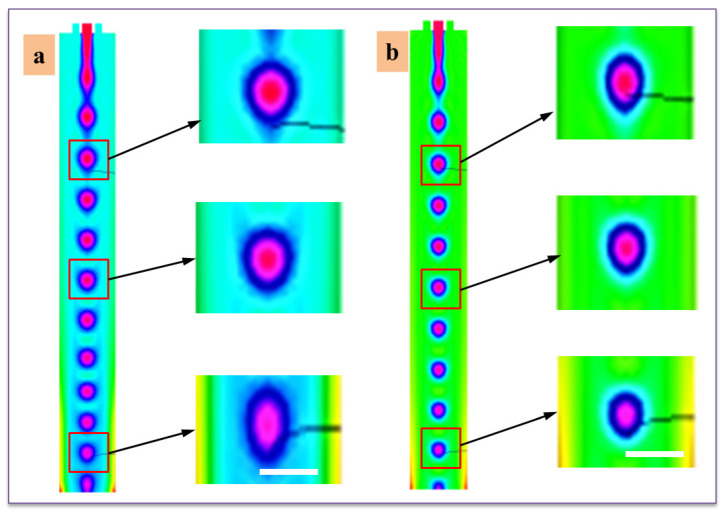
Representative droplet structures at shell/core flow velocity of (**a**) 150 m/s and dc pulse voltage of (**b**) 8.0 kV. Red color represents core fluids, blue color represents shell fluids, and green color is the surrounding air. Scale bar = 20 µm.

However, the internal flow velocity is limited by the minimum flow that the syringe pump can provide. Similarly, when fluid flow velocity is much less than a certain value (150 m/s), then fluid supplied by the syringe pump is unable to reach and maintain stability of the Taylor cone. This is because the reducing fluid flow velocity of the inner fluid causes liquid outflow to decrease, resulting in a reduction in diameter and length of the DoD CE-Jet. This will also reduce the feature size of the printed structure afterwards. The print height and applied pulse voltage have a similar impact on the DoD CE-Jet, and to actuate indirect changes in size and distribution of electric field. Throughout the simulation experiments, when the print height usage is too high, it is not enough to overcome surface tension with viscous force. Equally, fluid accumulated at the needle tip is larger than fluid sprayed to the silicon substrate making the DoD CE-Jet unstable. Likewise, low print height will generate large tangential electric field force, which prints small-size microdroplets. However, when print height is further reduced, then the micro-dripping mode will become unstable even though it causes electric field breakdown and droplet impairment. The representative compound droplet structures of simulations are presented at optimized values (150 m/s and 8.0 kV) as shown in Figure 8 with scale bar (20 µm).

### 3.4. Printing of Microdroplets Array

**Figure 9:**
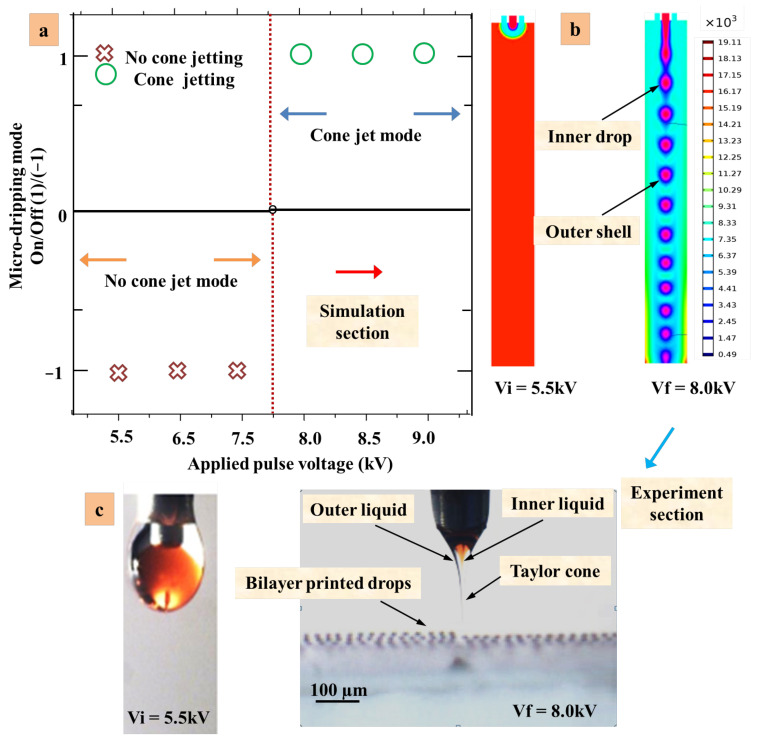
The contrast between simulation jetting and printing experimental jetting: (**a**) cone jetting status, (**b**) simulation results under dc pulse voltage, (**c**) the experimental results under identical parameters.

In general, the DoD CE-Jet printing approach is based on the micro-dripping mode when pulse voltage is applied, then it produces micro-dripping mode which is capable of printing bilayer micro/nano-sized dots. Similarly, continuous CE-Jet printing originates from the cone-jetting regime which can be obtained by applying DC voltage to the nozzle system to fabricate bilayer linear micro/nano structures such as nanowires and beams. In order to verify the simulation model, a series of experimental investigations were conducted using dc pulse voltages of 5.5 to 9.0 kV. It can be observed from simulation results that actual diameter of DoD CE-Jet is inversely proportional to pulse voltage, and the print height and the internal fluid flow velocities are in proportion. The size of the printed result is also in accordance with this rule, which proves correctness of the model and simulation results. Similarly, to verify correctness of the simulation results, we performed a series of experiments using two different liquids of PZT solution and photoresist (AZ703) as internal liquids. Moreover, high viscosity silicone oil was selected as the external fluid for comparative analysis. The PZT solution is an important piezoelectric material with high dielectric constant, high relative dielectric constant, and high electromechanical coupling. The advantages of combination coefficient and its nano-scale array line are the ideal structures for production of micro-strength sensors. An important family of functional materials is piezoelectric and therefore commonly called polar materials for M/NEMS devices. Subsequently, PZT and photoresist (AZ703) microscale droplets were printed by the DoD CE-Jet technique using optimized working parameters obtained from the phase field model. Figure 9 shows the contrast between simulation jetting and printing experimental jetting.

The experimental and simulation results are efficiently printed throughout this work. Figure 9a shows a comparison of cone jetting at the lowest and highest pulse voltage values. Thus, there is no cone jetting phenomenon at low voltages of 5.5 kV in the simulation study. Similarly, it can be seen from Figure 9b that when electric potential is low around the needle tip, then the cone jet is no longer. The coaxial cone jet forms a hemisphere when an electric field is applied around the needle tip. Thus, when applied voltage is set to 8.0 kV, then the PZT solution at the needle apex gradually develops droplet bubbles under the influence of a comprehensive electric force. Throughout the DoD CE-Jet printing method, two-layer microdroplets were produced in the needle vortex surrounded by air boundaries. Likewise, Figure 9c demonstrates experimental photographs taken with identical simulation parameters, which explains the cone-jetting mode at low and high electric potential around the needle tip. Subsequently, compound microdroplets were properly heated to 220 °C for 25 min to accelerate the evaporation of organic solution and solidification of the layers. If the temperature of the heating process is raised to 270 °C for 32 min, then it enhances the bond strength between inner droplet and substrate surface. Similarly, it can also prevent washing of microdroplets during removal of external silicone oil. The microdroplet generated by the PZT material has the same coherent frequency on the substrate due to strong effect of electric field with respect to outside air pressure. In the end, the silicon wafer was cautiously placed in isopropyl alcohol to remove the encapsulated outer shell of silicon oil when the substrate was cooled to room temperature. The microscale PZT droplets were taken on the substrate surface after removal of remaining liquid by heating the droplet array at 150 °C for 15 min in the ultrasonic cleansing machine. The conductivity of the functional ink is determined based on specific parameters that played a very important role in increasing the charge distribution effect in this study. The PZT and silicone oil are composed of different additives and their conductivity depends on the physical properties, type, and content of the additives. Moreover, the high-quality conductive functional ink meets several basic requirements in this work: (i) good stability, which ensures that the components do not decompose and aggregate easily; (ii) good rheological properties that ensure smooth printing on various substrates; (iii) compatibility with insulating substrates; (iv) low electrical resistance after annealing and film formation; (v) high-resolution printed microstructures; and (vi) low conversion temperature of ink to conductive film.

**Figure 10:**
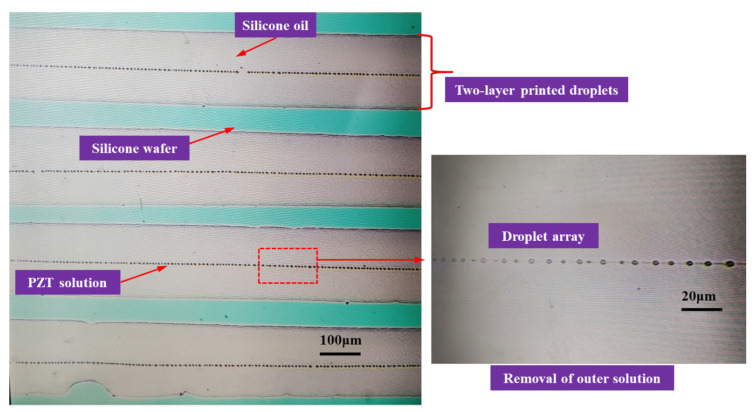
The DoD CE-Jet compound microdroplets structures on silicon substrate at optimized simulation parameters.

The main purpose of photoresist (AZ703) was to enhance the stability of droplets and further to increase coefficient of correctness of optimal printing parameters in the phase field simulation model. Based on the unique properties of photoresist (i.e., coated thickness range of 0.75–2.0 µm, recycled in dry and wet etch process environments, compatible with g-line and i-line, used in broadband exposure tools), it was used inside the inner needle by joining the inner liquid. It can be observed collectively in red color during simulation and experiment results. The optimized parameters are used (i.e., inner liquid flow velocity 150 m/s, pulse voltage 8.0 kV, external fluid velocity 250 m/s) for printing stable compound droplets. By applying these parameters, the study concludes that a stable CE-Jet micro-dripping regime is capable of printing stable compound microdroplets as shown in Figure 9c. The study displays that the simulated result of CE-Jet matches very well with the experimental results, which proves the correctness of the phase field coaxial model. This further illustrates that simulation of phase field can help to optimize DoD CE-Jet parameters and obtain desired microdroplets for the application of flexible printed electronics and micro/nano 3D printing structures. It has been previously mentioned that the photoresist and PZT solution was considered exceptional as an inner liquid for printing on a silicone substrate. Figure 10 shows DoD CE-Jet compound microdroplet structures on silicon substrate using optimized simulation parameters. Thus, a set of parallel droplet arrays are printed at a distance of 1.5 mm. The peculiarity of the size of printed PZT microdroplets was determined at 20 µm inside the outer solution. The minimum diameter of the microdroplets after removal of the outer solution measured ~7.5 µm along with standard deviation and the gap of the microdroplets was maintained at a distance of 20 µm as shown in Figure 10. The experimental results exhibit that droplets printed by PZT solution were very uniform and condensed inside the outer shell of silicone oil. Furthermore, the applied voltage was too high, which actuated immediate and packed electric field effects around the coaxial needle. Thus, due to the strong effect of electric field, the residue charges were reduced on the substrate surface.

**Figure 11:**
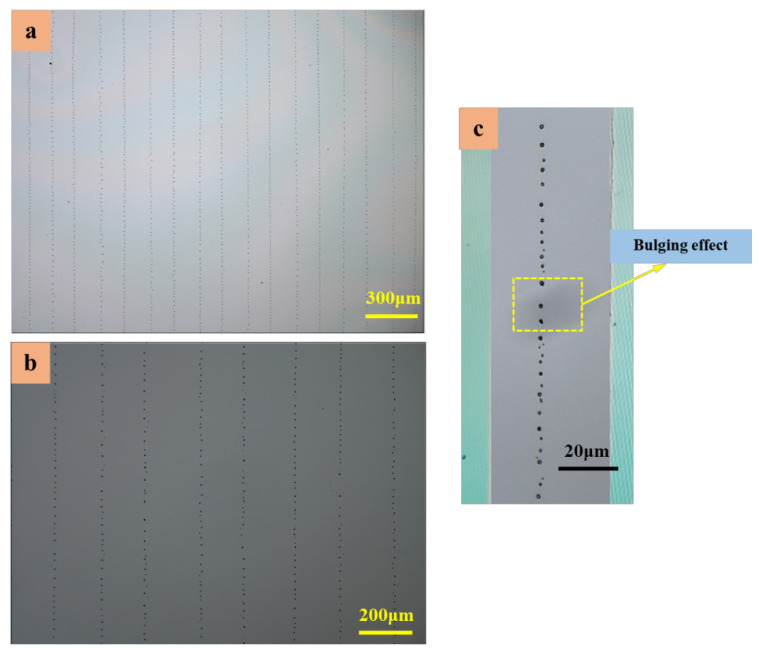
The printed droplets under different process parameters; (**a**,**b**) analysis of the coffee ring effect on the printed microdroplets keeping different distances between two dots; (**c**) the deposited microdroplets line on silicon substrate generating bulging effect.

Figure 11 demonstrates the stability and morphology of the droplets formed on the silicon substrate under different applied voltages. The coffee ring effect is induced when the applied voltage is below 8.0 kV, keeping the same parameters. Figure 11a shows that the printed dots are unstable and random because the distance between consecutive ones is small (2 µm), which caused the main instability behavior. Moreover, Figure 11b shows that the coffee ring effect is not higher due to the large gap (4 µm) between the printed arrays and the gap between the two dots. Figure 11c shows the random line pattern of compound droplets using high-pulse voltage. The micro-dripping mode is not in sequence due to the bulging effect on the inner liquid, where the outer morphology of printed droplets appears significantly inflated outward. However, the bulging effect was small in the droplets array due to considerable selection of applied parameters in experiments. Similarly, the directed movement of substrate in the XYZ rotation stage did not disrupt morphology of PZT droplets on the inner part of the outer shell. The minimum diameter of droplets after removal of the outer solution was measured ~5.5 µm after standard deviation. This concludes that DoD CE-Jet technique is capable of producing highly stable and control droplets for complex structures in electronic devices. The experimental and numerical work was carefully carried out and then the droplet size was measured in both the simulation and the experiment case to check the accuracy of the simulation results. The simulated droplet size was measured using ImageJ software, which shows that the minimum bilayer droplet size was 18 µm. However, when the outer shell of the droplet is not included, then the inner size of the droplet was about 6 µm, which proves that the experimental results almost match the simulated results. Finally, the printed microstructures prove the correctness and accuracy of the simulation model.

## 4. Conclusions

In this paper, a phase field DoD CE-Jet simulation model is established which controls the influence of key printing parameters on cone jet morphology and droplet diameter. The coaxial needle shape and relative length of the needle are designed and manufactured according to the DoD CE-Jet process. The coaxial printing experiments were carried out using the photoresist and PZT sol in the inner needle and silicone oil in the outer needle, respectively, which verified the correctness of the simulation results. Therefore, the establishment of a hydrodynamic force regime for a coaxial electric jet, the equation derived from internal fluid motion, and the electric field equation were added to the Navier–Stokes equation. Further, the Maxwell pressure tensor was set up to trace the interface equation between two fluids. The simulation results revealed that the diameter of the inner jet decreases with increasing applied pulse voltage. Likewise, the diameter of the inner jet was proportional to the flow velocity and print height. For the effect of morphology near needle tip, it was found that the diameter of the CE-Jet increases with increasing print height. To verify simulation results and to guide the printing experiment, the PZT sol and photoresist (AZ703) were selected as the inner liquid. The experimental results showed that when no electric field is applied, the liquid volume is concentrated in a spherical shape. Similarly, once the electric field is set, a stable CE-Jet can be formed with a microscale inner jet, which preliminarily verifies the reliability of the simulation. The silicon oil microscale line with a size of 105 µm and PZT microdroplets with a minimum size of ~5.5 µm were printed directly under the optimized parameters obtained from the simulation. Thus, it is proved that droplet generation is inversely proportional to pulse voltage and directly proportional to internal liquid flow velocity and print height. Overall, this simulation work demonstrates that high pulse voltage and low flow velocity can be used to fabricate excellent features with potential applications in M/NEMS devices. This method is low in cost, easier to handle, and requires less time to use in E-Jet printing.

## Figures and Tables

**Table 1:** PZT solution, photoresist AZ703, and silicone oil properties used for the DoD CE-Jet printing process.

Liquid Properties	Density *ρ* (kg·m^−3^)	Viscosity µ (Pa·s)	Surface Tension Coefficient σ (N·m^−1^)	Relative Permittivity (ɛ)
Photoresist (AZ703)	1050	1.4 × 10^−2^	2.83 × 10^−2^	4.8
PZT solution	1061	1.6 × 10^−2^	1.93 × 10^−2^	22
Silicone oil	976	58.56	22	2.77

**Table 2:** The boundary conditions of the geometric model.

Boundary	Electrostatic Field	Hydrodynamic Field
A: Inner needle inlet	*φ = V_0_*	*u = Q_inner_/A_inner_*
B: Outer needle inlet	*φ = V_0_*	*u = Q_outer_/A_outer_*
C: Wall of inner needle	*φ = V_0_*	*u = 0*
D: Wall of outer needle	*φ = V_0_*	*u = 0*
E: axisymmetric space	*φ_r_ = 0*	*u_r_ = 0*
F: Boundary of computational territory	*φ = V*	*P = 0*
G: Outlet of coaxial needle	*φ = 0*	*P = 0*

## Data Availability

Not applicable.
